# Piloting an integrated SARS-CoV-2 testing and data system for outbreak containment among college students: A prospective cohort study

**DOI:** 10.1371/journal.pone.0245765

**Published:** 2021-01-26

**Authors:** Laura Packel, Arthur Reingold, Lauren Hunter, Shelley Facente, Yi Li, Anna Harte, Guy Nicolette, Fyodor D. Urnov, Michael Lu, Maya Petersen, on behalf of the IGI Testing Consortium

**Affiliations:** 1 Division of Epidemiology and Biostatistics, School of Public Health, University of California Berkeley, Berkeley, California, United States of America; 2 Facente Consulting, Richmond, California, United States of America; 3 University Health Services, University of California Berkeley, Berkeley, California, United States of America; 4 Innovative Genomics Institute, University of California Berkeley, Berkeley, California, United States of America; Mansoura University, EGYPT

## Abstract

**Background:**

Colleges and universities across the country are struggling to develop strategies for effective control of COVID-19 transmission as students return to campus.

**Methods and findings:**

We conducted a prospective cohort study with students living on or near the UC Berkeley campus from June 1st through August 18th, 2020 with the goal of providing guidance for campus reopening in the safest possible manner. In this cohort, we piloted an alternative testing model to provide access to low-barrier, high-touch testing and augment student-driven testing with data-driven adaptive surveillance that targets higher-risk students and triggers testing notifications based on reported symptoms, exposures, or other relevant information. A total of 2,180 students enrolled in the study, 51% of them undergraduates. Overall, 6,247 PCR tests were administered to 2,178 students over the two-month period. Overall test positivity rate was 0.9%; 2.6% of students tested positive. Uptake and acceptability of regular symptom and exposure surveys was high; 98% of students completed at least one survey, and average completion rate was 67% (Median: 74%, IQR: 39%) for daily reporting of symptoms and 68% (Median: 75%, IQR: 40%) for weekly reporting of exposures. Of symptom-triggered tests, 5% were PCR-positive; of exposure-triggered tests, 10% were PCR-positive. The integrated study database augmented traditional contact tracing during an outbreak; 17 potentially exposed students were contacted the following day and sent testing notifications. At study end, 81% of students selected their desire “to contribute to UC Berkeley’s response to COVID-19” as a reason for their participation in the Safe Campus study.

**Conclusions:**

Our results illustrate the synergy created by bringing together a student-friendly, harm reduction approach to COVID-19 testing with an integrated data system and analytics. We recommend the use of a confidential, consequence-free, incentive-based daily symptom and exposure reporting system, coupled with low-barrier, easy access, no stigma testing. Testing should be implemented alongside a system that integrates multiple data sources to effectively trigger testing notifications to those at higher risk of infection and encourages students to come in for low-barrier testing when needed.

## Introduction

Colleges and universities across the country are struggling with the design of strategies for effective control of COVID-19 transmission. Multiple institutions have attempted to resume on-campus learning, only to reverse course due to COVID-19 outbreaks among students [1, 2]. At many universities, students have been blamed for what was considered to be irresponsible behavior [3, 4], and suspended or threatened with fines or expulsion [5–8]. While some have advocated for a harm-reduction approach to student engagement [9], best practices for doing so remain to be defined.

The design of COVID-19 outbreak prevention and detection systems offers an important opportunity to engage students as partners in COVID-19 outbreak control; however, to date this potential remains to be realized on most campuses. Testing strategies for symptomatic or exposed students often rely on traditional student health service delivery approaches, which require phone-call based screening and appointment scheduling. Traditional contact tracing on university and college campuses in the United States similarly remains largely reliant on phone calls or in-person interviews and requires sufficient trust to elicit contact information. Among a student population that relies heavily on text-driven communication and may fear judgement or disciplinary reprisal for reporting risky behaviors, such approaches may introduce barriers to rapid detection and isolation of infected individuals.

Some universities have attempted to address these barriers via frequent asymptomatic testing programs [10, 11], such as mandating testing for all students twice weekly (a frequency suggested by modeling to provide a secure path to outbreak containment) [11]. However, such approaches also have significant drawbacks. For many universities such uniform high intensity testing strategies may be logistically, legally, and financially unfeasible, particularly when large numbers of students live in off-campus housing.

Improved low barrier testing approaches that are better able to leverage student initiative and cross-campus partnerships while integrating data from multiple sources are thus needed as colleges and universities navigate the COVID-19 pandemic. Between June and August 2020, we developed and evaluated a testing and response system as part of the Berkeley Safe Campus Initiative, a multidisciplinary partnership between the University of California, Berkeley School of Public Health, University Health Services (UHS), and the Innovative Genomics Institute. In this brief report, we present key elements of this student-friendly, adaptive, data-driven testing model and discuss lessons learned that are relevant for colleges and universities across the country as they grapple with how to resume student life on campus in the context of COVID-19.

## Materials and methods

### Design of the safe campus system

**Fig 1:**
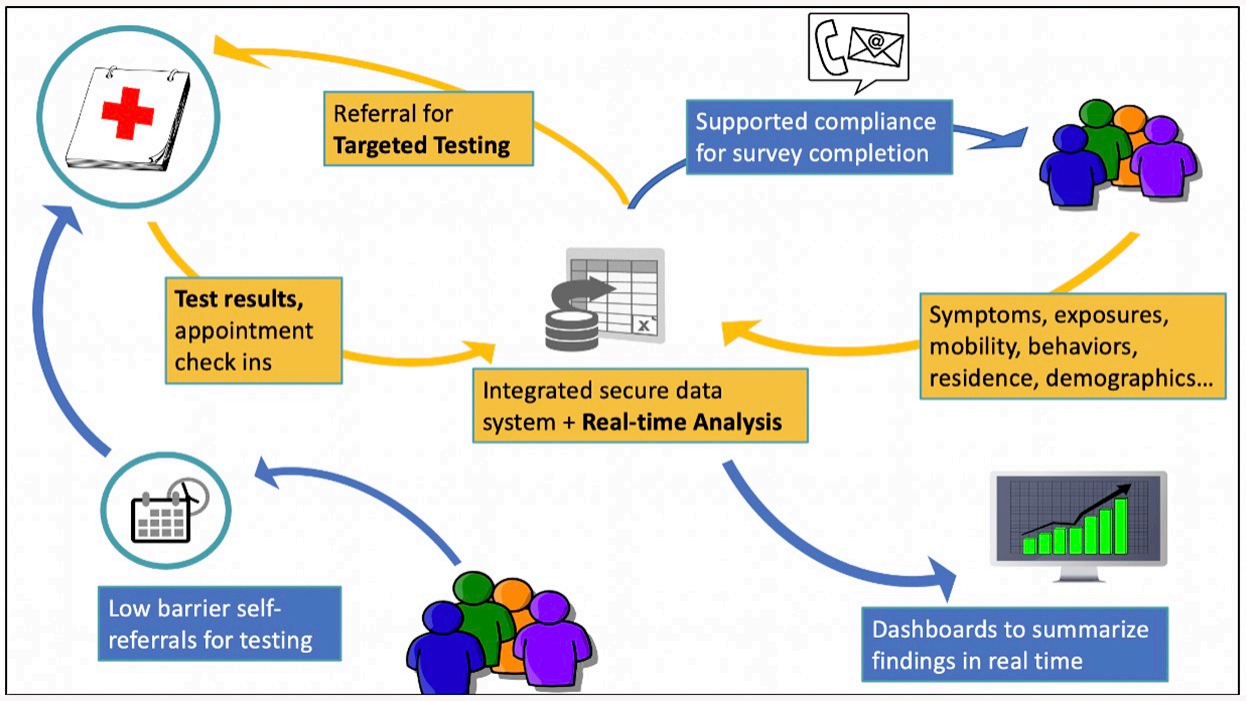
Safe campus study integrated data system.

The Berkeley Safe Campus Initiative was designed as an innovative outbreak detection and prevention system based on an integrated database for monitoring and responding to risk on campus. The database contains voluntary, confidential and regularly updated information on students’ households, behaviors, symptoms, and exposures. Through a partnership with UHS, the database was linked to all PCR test results for students enrolled in the study. The Safe Campus system was designed to enable timely detection of and response to possible COVID-19 clusters and to enable smart “adaptive” triggers for surveillance testing. Core elements (Fig 1) included: 1) a low-barrier “zero stigma” online appointment system; 2) confidential regular device-based symptom and exposure screening, linked to automated testing and quarantine notifications; and 3) rapid location-based surveillance testing notifications to augment contact tracing through UHS. Each component was designed to engage students as partners in outbreak detection and containment.

#### Low-barrier self-scheduling for PCR testing

An online appointment system was provided for easy self-scheduling of tests without consulting with a healthcare provider. All testing was provided without cost to students. The system was designed to remove both logistical barriers to testing (e.g. the hassle and inefficiency of phone calls) and psychosocial barriers (e.g. fear of disciplinary consequences, negative judgement, or accusations for engaging in risk behaviors). This type of scheduling offered students a frictionless means to act on their own self-assessed risk for infection, including using information shared through student social networks and online social media platforms. The ability to schedule and attend testing together with friends and housemates provided an additional means to positively leverage peer perceptions, norms and behaviors and enable testing uptake [12, 13].

#### Regular symptom and exposure screening linked to triggered testing

Students were asked to complete daily web-based surveys reporting symptoms and subjective concern for risk of COVID19, and weekly web-based surveys on known or suspected exposures. Participation in surveys was voluntary, confidential, and, unlike symptom screeners currently in use on many campuses, was not tied to campus building access, legal action, or potentially negative consequences. Students received a single $50 gift card after completing a 30-minute baseline survey, baseline testing, and their first 10 daily surveys; this incentive structure was designed as both a nudge to encourage participation and a means to facilitate habit formation for daily survey completion [14–16].

Report of certain symptoms (i.e., temperature ≥100.4°F, feeling feverish, dry cough, coughing up mucus, unusual pain or pressure in the chest, difficulty breathing, shortness of breath, unexplained trouble thinking or concentrating, loss of sense of taste or smell) or of recent contact with a household member with these symptoms and/or who was suspected or confirmed to have COVID-19 triggered an automated notification within the survey and a reminder email. The notification provided information on quarantine, an encouragement to test immediately, and a link to scheduling testing via the online “no-questions asked” system. In addition to triggering testing, surveys aimed to maintain student engagement with campus as partners in outbreak control. To facilitate open communication with students, study staff were available to answer questions via phone, text, and email 12 hours per day.

#### Augmented rapid contact notification

On entry to the system, detailed contact information (residential address, phone number, and email) and survey data on household characteristics and risk factors were collected. Because all test results were linked back to the Safe Campus database, rapid augmented contact notification for students with PCR results positive for SARS-CoV-2 was facilitated. For example, device-based testing notifications, including quarantine guidelines, could be sent immediately to all students sharing a residence with a newly identified case, a particularly important capability in a campus setting where 73% of undergraduate students live in non-university affiliated housing, which is often high density. Further, in the context of an emerging outbreak, likely contact notifications could be expanded to include students reporting shared risk factors (such as Greek affiliation) or behaviors (such as attendance at large social events). This system avoided delays in contact identification and notification that often occur with traditional contact tracing (serial interviews and phone calls), and aimed to strengthen student engagement by providing two way communication, giving students the ability to text, email, or call to immediately reach study staff who could provide instructions for accessing testing and quarantine information.

### Study population and measures

Between June 1, 2020 and August 18, 2020, we conducted a pilot study to evaluate this Safe Campus system among students enrolled at the University of California, Berkeley. Inclusion was limited to students with plans to reside in one of the two counties nearest to campus (Alameda and Contra Costa) over the course of the summer. In addition to survey data integral to the implementation of the Safe Campus system (including daily symptom and weekly exposure questionnaires), we collected endline survey data, including free text responses, on student perceptions, attitudes, and behaviors related to outbreak control. Survey data were collected and stored in an integrated REDCap [17, 18] data system.

Nasal swabs for SARS-CoV-2 testing were collected by UHS staff at outdoor tents on campus; UHS also led all contact tracing and clinical management of PCR+ cases identified during the study. PCR testing for SARS-CoV-2 was performed on campus by the Innovative Genomics Institute [19], enabling turnaround times generally <48 hours during a period when there were substantial delays regionally. In addition to PCR tests triggered by the Safe Campus system, additional comprehensive PCR testing was conducted at study baseline and endline for research purposes. This study was approved by the University of California, Berkeley Committee for the Protection of Human Subjects (protocol #2020-05-13261), all study participants were required to provide written, informed consent.

## Results

**Fig 2:**
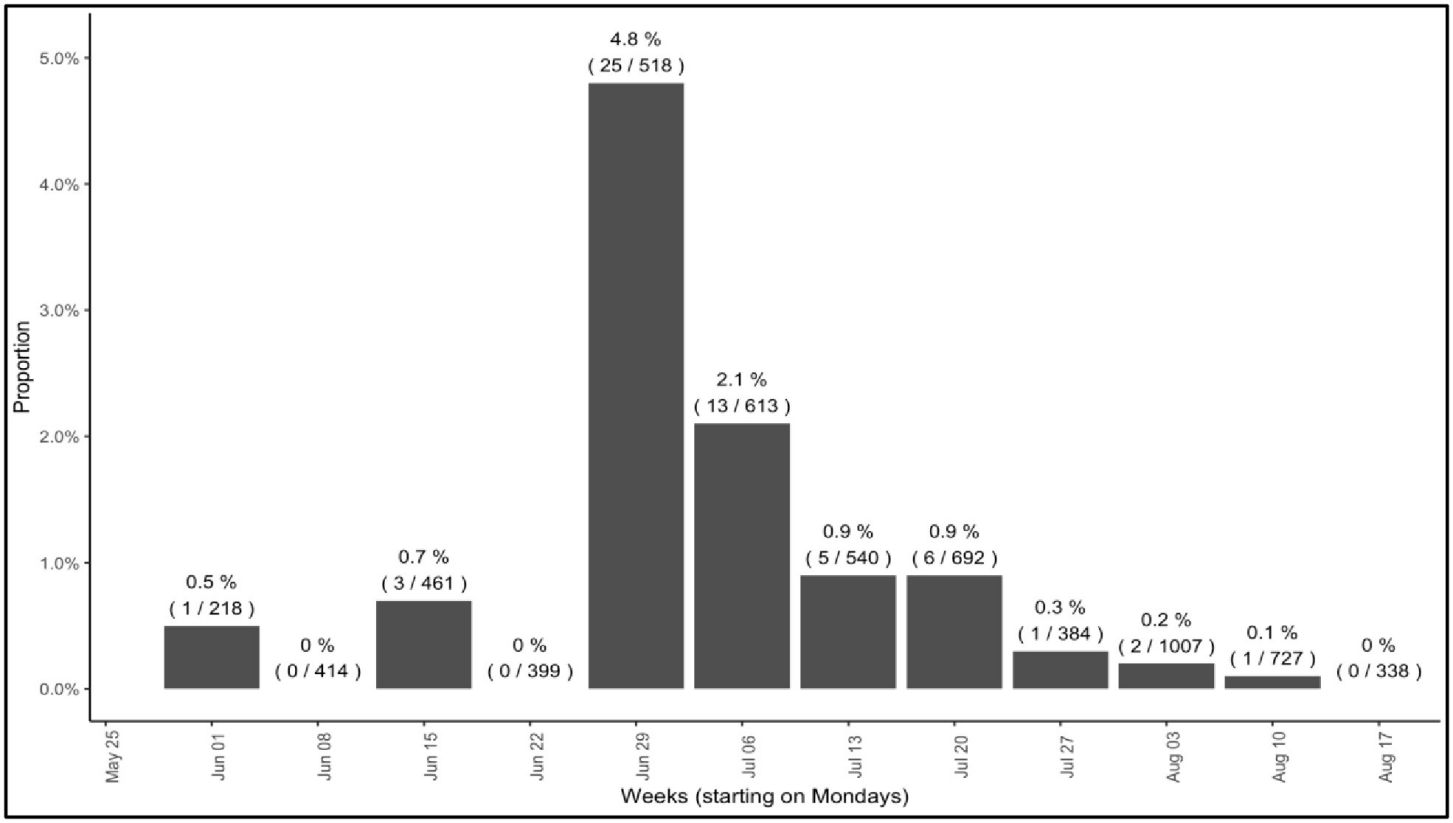
COVID-19 test positivity by week.

**Fig 3:**
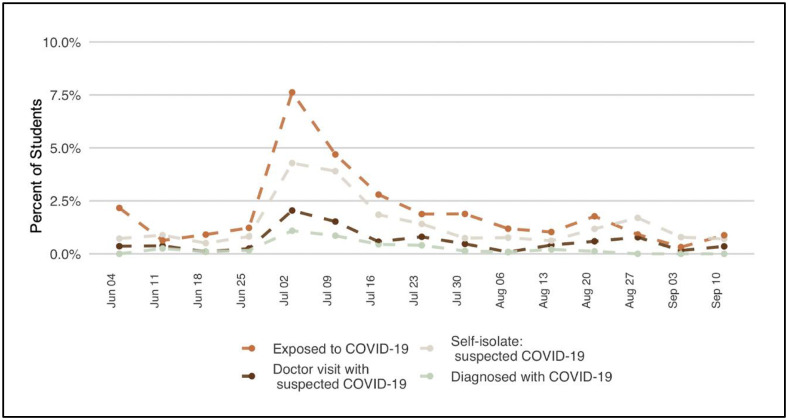
COVID-19 experiences by week, as reported in weekly exposure surveys.

**Fig 4:**
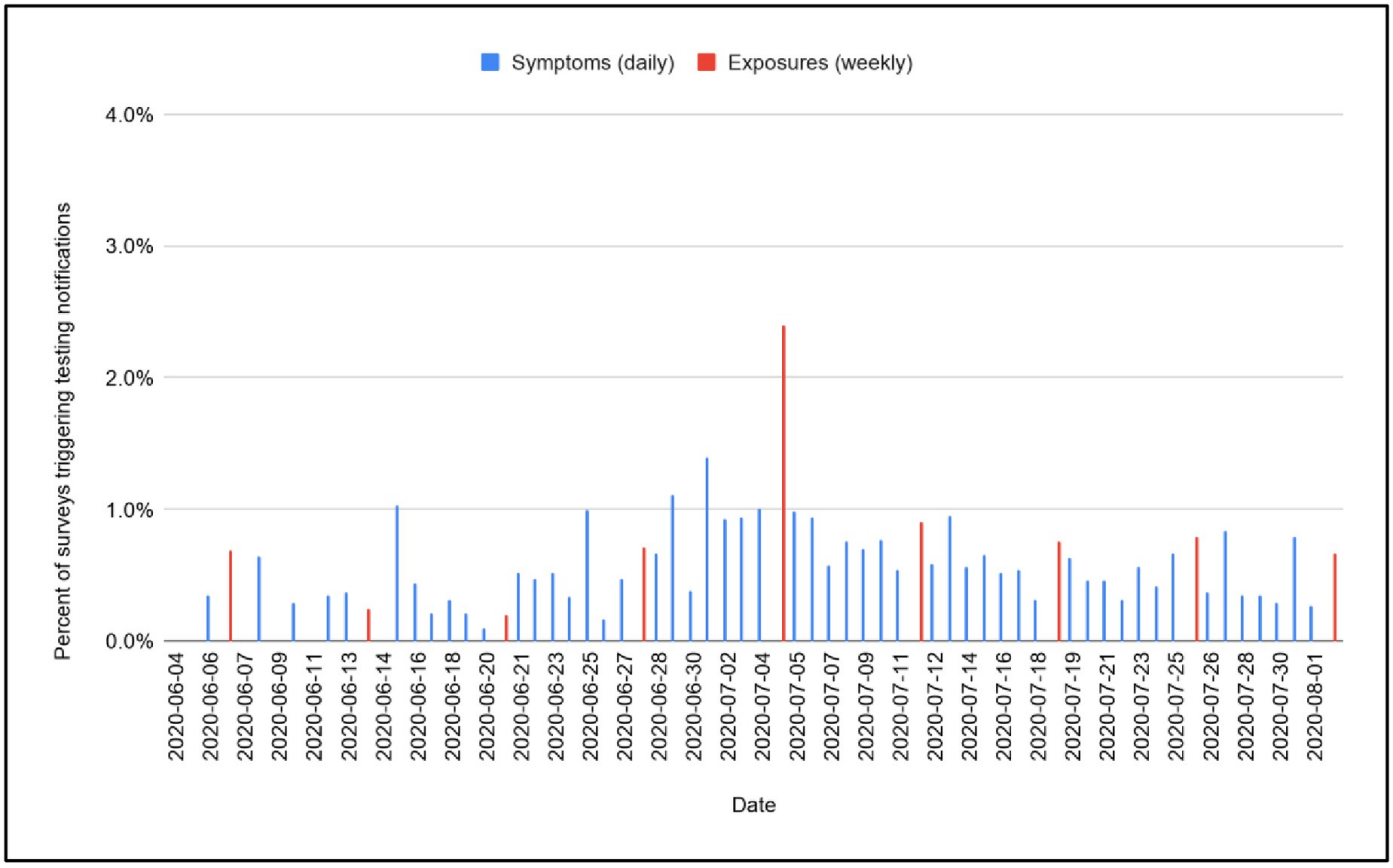
Proportion of daily/weekly surveys that triggered testing notifications.

A total of 2,180 students enrolled in the summer pilot, 51% of them undergraduates. Overall, 6,292 PCR tests were administered to 2,177 students over the two-month period. Overall test positivity rate was 0.9%; 2.6% of students tested positive. A cluster of 25 cases emerged in the four days prior to the July 4th holiday weekend, centered among students living in Greek housing, but subsequently including cases among persons who had attended shared social events. During that week, test positivity peaked at 4.1% (Fig 2). While reported symptoms and exposures were infrequent throughout the study period, the proportion of students reporting exposure to COVID-19, the proportion reporting self-isolation because of suspected exposure (Fig 3), and the proportion of surveys that triggered a testing notification due to reported symptoms or exposures (Fig 4) all increased during the outbreak week in July.

### Low barrier testing system to leverage social networks

The emerging outbreak, which coincided with an email promotion of the study to all Greek-housed students, was followed by changes in testing patterns among students in Greek housing. As early word of the COVID-19 outbreak spread through social networks, 90 students living in Greek housing enrolled in the Safe Campus system and scheduled a test–a 718% increase in enrollment among Greek-housed students compared to the previous week. Timing of testing appointments was highly clustered by house of residence, strongly suggesting that students made and attended appointments with social groups; of 95 testing appointments completed by Greek-housed students during the week of the outbreak, 56% took place within 10 minutes of another student living in the same Greek house.

### Regular symptom and exposure screening to trigger testing

Uptake and acceptability of regular symptom and exposure surveys was high; 98% of students completed at least one survey, and average completion rate was 67% (Median: 74%, IQR: 39%) for daily reporting of symptoms and 68% (Median: 75%, IQR: 40%) for weekly reporting of exposures. At the end of the study, nearly 85% of students stated they would be “likely” or “extremely likely” to continue to participate in the daily and weekly surveys should campus continue them beyond the study. Students also made extensive use of the open communication with the study team; the study received over 1,300 emails and over 1,200 text messages and phone calls over the course of the study. During the two months of follow-up, 373 students reported symptoms and 177 reported exposures that triggered a testing notification; of these, 260 (70%) and 90 (51%) students, respectively, completed a PCR test at UHS within a week. Among symptom-triggered tests, 8% (23/299) were PCR-positive; among exposure-triggered tests, 8% (8/102 were PCR-positive. For comparison, overall test positivity during the study was 0.9% (inclusive of persons self-referring into the study for baseline testing in the context of the outbreak).

### Augmented contact tracing

Use of residential and household information from the integrated database enabled rapid contact notifications early in the course of the outbreak, augmenting traditional contact tracing. Following detection of three cases among residents in a Greek house on June 30th, testing notifications were emailed on July 1st to 17 other participants in Greek housing who did not already have a recent or pending test result. As additional cases were detected among students outside of Greek housing, testing notifications were expanded to include co-op-housed participants and those living at other addresses with identified case(s). This process augmented and accelerated contact tracing based on telephone contact and individual interview with index cases. During the outbreak, 44 students were sent testing notifications using this system; 33 came in for testing within the following week, one of whom tested positive for COVID-19.

### Student attitudes towards partnering with campus

At endline, 79% of students selected their desire “to contribute to UC Berkeley’s response to COVID-19” as a reason for their participation in the Safe Campus system. In addition, given the opportunity and the right environment, students were ready and willing to test regularly; 88% stated they were likely or very likely to comply with regular testing, with a preference for saliva-based testing on a weekly basis. Finally, endlike comments reflected positive perceptions of the Safe Campus system:

*Testing was made so easy and accessible. Even having the option of readily-available testing made a world of a difference*.

*Having the ability to get easily tested made me feel much more secure! Please, if you have any power to do so, ensure that campus students can be swab tested on demand as often as desired*.

*Thank you for conducting this study. I was proud to participate in research that will hopefully put the campus on fronted footing to implement evidence-based protocols for the upcoming semesters*.

## Discussion

Our study highlights an alternative testing model that is student-centered; provides access to low-barrier, high-touch testing; and augments student-driven testing with data-driven adaptive surveillance testing, triggering testing notifications based on reported symptoms, exposures, or other relevant information. Pilot results provide initial proof-of-concept for the viability of the Safe Campus approach to effectively target testing to students potentially at risk of infection. Our end-of-study data indicate that students want to help and to contribute to efforts to keep campus safe.

While a promising start, the Safe Campus system described here has a number of limitations. First, not all students in the area were enrolled in the study. While greater coverage of the student population would be expected to contribute further to outbreak control, it is likely that the students who chose to enroll in the study were also those more positively engaged with COVID-19 outbreak control efforts. Second, the data system only captured results of PCR tests from UHS testing; however, we believe testing outside the free low barrier option offered through the study was uncommon. Third, the type of incentives used in this study may not be feasible at scale; however, there are other non-monetary nudges that could motivate participation in regular symptom or exposure surveys, such as leveraging social norms or commitments [14, 15].

The Safe Campus system described here could be improved in future iterations. First, additional data sources, potentially including wastewater-based monitoring [20, 21] and app-based mobility and contact data, could be integrated to improve risk assessment. Second, the Safe Campus database could be linked to de-identified regional health department data to further improve reach and more directly inform regional public health initiatives. Third, in settings and periods with higher test positivity rates, online machine learning algorithms could be used to provide time-updated risk stratification for test targeting [22]. Finally, effective testing strategies will contribute to outbreak containment only if coupled with rapid and effective isolation for persons with PCR results positive for SARS-CoV-2. Recent experiences on university campuses illustrate the additional challenges posed by this step in the COVID-19 infection control cascade [23].

In summary, the Safe Campus Initiative illustrates the synergy created by bringing together a student-friendly, harm reduction approach to testing and integrated data/analytics. We recommend the use of a confidential, consequence-free, incentive-based daily symptom and exposure reporting system, coupled with low-barrier, easy access, no stigma testing and personalized support for students who test positive. Critically, this testing environment should be implemented alongside a data system that integrates multiple data sources to effectively trigger testing notifications to those at higher risk of infection and encourages students to come in for low-barrier testing when needed. In our experience, providing on-demand, no stigma testing, coupled with easy-access communication via text and email bolsters students’ confidence that the campus is doing everything they can to keep students safe, and importantly, empowers them to contribute significantly to a safer campus during the COVID-19 pandemic.
